# Brd4 BD1 Domain Antagonism of MS436 Preserves Blood‐Brain Barrier Integrity via Rnf43/β‐Catenin Signaling Pathway

**DOI:** 10.1002/advs.202515584

**Published:** 2025-11-20

**Authors:** Chenxiao Li, Xiaochen Zhang, Jiangwei Xia, Shuang Liu, Yushan Du, Sihan Li, Shukui Zhang, Yong Zhang, Fen Ji, Jianwei Jiao, Jingjing Zhang

**Affiliations:** ^1^ Zhanjiang Key Laboratory of Zebrafish Model for Development and Disease Affiliated Hospital of Guangdong Medical University Zhanjiang 524001 China; ^2^ Key Laboratory of Organ Regeneration and Reconstruction Chinese Academy of Science Beijing 100101 China; ^3^ University of Chinese Academy of Sciences Beijing 100049 China; ^4^ Academy of Medical Engineering and Translational Medicine Tianjin University Tianjin 300072 China; ^5^ Department of Neurology Xuanwu Hospital Capital Medical University, National Center for Neurological Disorders Beijing 100053 China; ^6^ Jiaozuo Hospital of Traditional Chinese Medicine Jiaozuo 454150 China; ^7^ College of Basic Medicine Qingdao University Qingdao 266071 China; ^8^ Neuroscience Research Institute and Department of Neurobiology, School of Basic Medical Sciences Peking University, Key Laboratory for Neuroscience, Ministry of Education of China and National Health Commission of the People's Republic of China Beijing 100083 China; ^9^ School of Medical Technology Guangdong Medical University Dongguan 523808 China

**Keywords:** blood‐brain barrier, bromodomain, cerebrovascular diseases, Claudin 5, tight junction

## Abstract

Blood‐brain barrier (BBB) disruption is central to neurodegenerative and cerebrovascular diseases, but its causal role and therapeutic targeting remain challenging. Bromodomain and extraterminal domain (BET) proteins, particularly Brd4, regulate transcription via dual bromodomains (BD1, BD2). While BD1 antagonists are explored for tumors and BD2 antagonists for inflammation, their impact on cerebrovascular integrity is unclear. Crucially, opposing BBB effects of domain‐specific BET antagonism: the BD2 antagonist RVX208 disrupt the BBB, whereas the BD1 antagonist MS436 significantly reduces leakage and improves neurological outcomes, revealing therapeutic potential is demonstrated. Mechanistically, endothelial Brd4 critically suppresses BBB stability. Its ablation upregulates tight junction (TJ) proteins. Brd4, acting exclusively through its BD1 domain, destabilizes TJs via Rnf43 is identified, which promotes β‐catenin and TJ protein degradation. This defines a novel Brd4 BD1/Rnf43/β‐catenin axis essential for BBB integrity. The findings establish Brd4 BD1 inhibition as a novel BBB‐protective strategy and position MS436 for repurposing in cerebrovascular diseases, necessitating reevaluation of domain‐specific BET targeting for neurovascular pathologies.

## Graphical abstract

MS436 competitively binds to the BD1 domain of Brd4, thereby suppressing Brd4 induced degradation of tight junction proteins via the Rnf43‐Fzd4‐β‐catenin signaling pathway. Consequently, this attenuation of degradation reduces blood‐brain barrier leakage, leading to an improved overall prognosis after stroke.

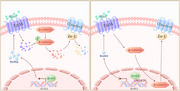

## 1 Introduction

The blood‐brain barrier (BBB) represents a highly specialized and selective barrier that segregates the central nervous system from the peripheral circulation. This sophisticated interface plays a crucial role in maintaining cerebral homeostasis by protecting the brain from potentially harmful blood‐borne substances while facilitating selective nutrient transport.^[^ ^1^ ^]^ BBB disruption is a well‐documented pathological feature in numerous neurodegenerative diseases, including Parkinson's disease (PD), Alzheimer's disease (AD), and multiple sclerosis (MS),^[^ ^2^ ^]^ as well as in acute cerebrovascular disorders such as stroke.^[^ ^3^ ^]^ Notably, multiple genetic risk factors associated with neurodegenerative diseases have been implicated in BBB disruption.^[^ ^4^, ^5^ ^]^ For instance, APOE4, the most significant genetic risk factor for sporadic AD, has been demonstrated to induce BBB dysfunction.^[^ ^6^ ^]^ Clinical studies revealed that APOE4‐mediated BBB disruption in the hippocampus correlates with cognitive decline, with APOE4 status serving as a predictive biomarker for the rate of cognitive deterioration.^[^ ^7^ ^]^ Similarly, amyloid precursor protein (APP), a key pathogenic factor in familial AD, has been shown to compromise BBB integrity in both experimental models^[^ ^8^ ^]^ and human studies.^[^ ^9^ ^]^ However, the precise role of BBB disruption in disease pathogenesis remains controversial, with ongoing debate about whether it represents a causative factor or a secondary epiphenomenon.

Bromodomains (BD), which are uniquely capable of recognizing and binding acetylated histones, play an indispensable role in transcriptional regulation.^[^ ^10^, ^11^ ^]^ Different from other family member that possess only one bromodomain, the BET (bromodomain and extraterminal domain) family represents a distinct subfamily characterized by the presence of two functionally diverse bromodomains (BD1 and BD2) with distinct downstream targets.^[^ ^11^, ^12^, ^13^ ^]^ The differential expression of BET proteins in cancers and inflammatory diseases has spurred extensive research into BET antagonists as therapeutic agents. Current therapeutic strategies have demonstrated domain‐specific applications: BD1‐targeting antagonists are primarily utilized in oncology,^[^ ^14^, ^15^ ^]^ while BD2‐selective antagonists show promise in anti‐inflammatory therapies.^[^ ^16^, ^17^ ^]^ Notably, BD2‐targeting antagonists have shown significant potential in cardiovascular disease treatment. For instance, the BD2‐specific antagonist RVX‐208 has been shown to reduce aortic lesions, modulate lipid profiles by increasing high‐density lipoprotein (HDL) and decreasing low‐density lipoprotein (LDL), and suppress inflammatory cytokine levels in *APOE* knockout mouse models.^[^ ^18^ ^]^ Additionally, BD2 inhibition or selective overexpression of Brd4's BD2 domain has been demonstrated to inhibit endothelial‐mesenchymal transition and attenuate neointimal formation in rat jugular vein graft models.^[^ ^19^ ^]^

Given these cardiovascular effects, we investigated BET inhibition in the brain, where the blood‐brain barrier (BBB) presents unique therapeutic challenges and opportunities. While BET antagonists have been explored in BBB‐disrupting diseases like AD^[^ ^20^, ^21^ ^]^ and stroke,^[^ ^22^, ^23^, ^24^ ^]^ attributed effects primarily stem from reduced inflammation. Crucially, domain‐specific BET antagonists remain unexplored for cerebrovascular BBB protection.

Strikingly, antagonists targeting different BET BDs exert opposing effects on tight junction (TJ) protein levels: BD1 inhibition upregulates TJs, whereas BD2 inhibition downregulates them. Based on this finding and its therapeutic potential, we focused on the BD1 antagonist MS436. MS436 effectively prevented BBB disruption and improved stroke outcomes. Mechanistically, Brd4 facilitates Rnf43 transcription; Rnf43 then downregulates the Wnt receptor Fzd4, reducing β‐catenin levels and promoting TJ protein degradation. Critically, Brd4 regulates TJs specifically via its BD1 domain. Consequently, MS436 treatment restores β‐catenin function and rescues TJ integrity.

Our study suggests that BBB‐protective therapeutics using MS436 represents a promising intervention for acute vascular disorders, particularly stroke. Furthermore, we propose that these pharmacological agents may hold therapeutic potential for chronic neurodegenerative diseases, including but not limited to AD, PD, and MS.

## 2 Results

### 2.1 MS436 Upregulates the Expression of Tight Junction Proteins

**Figure 1:**
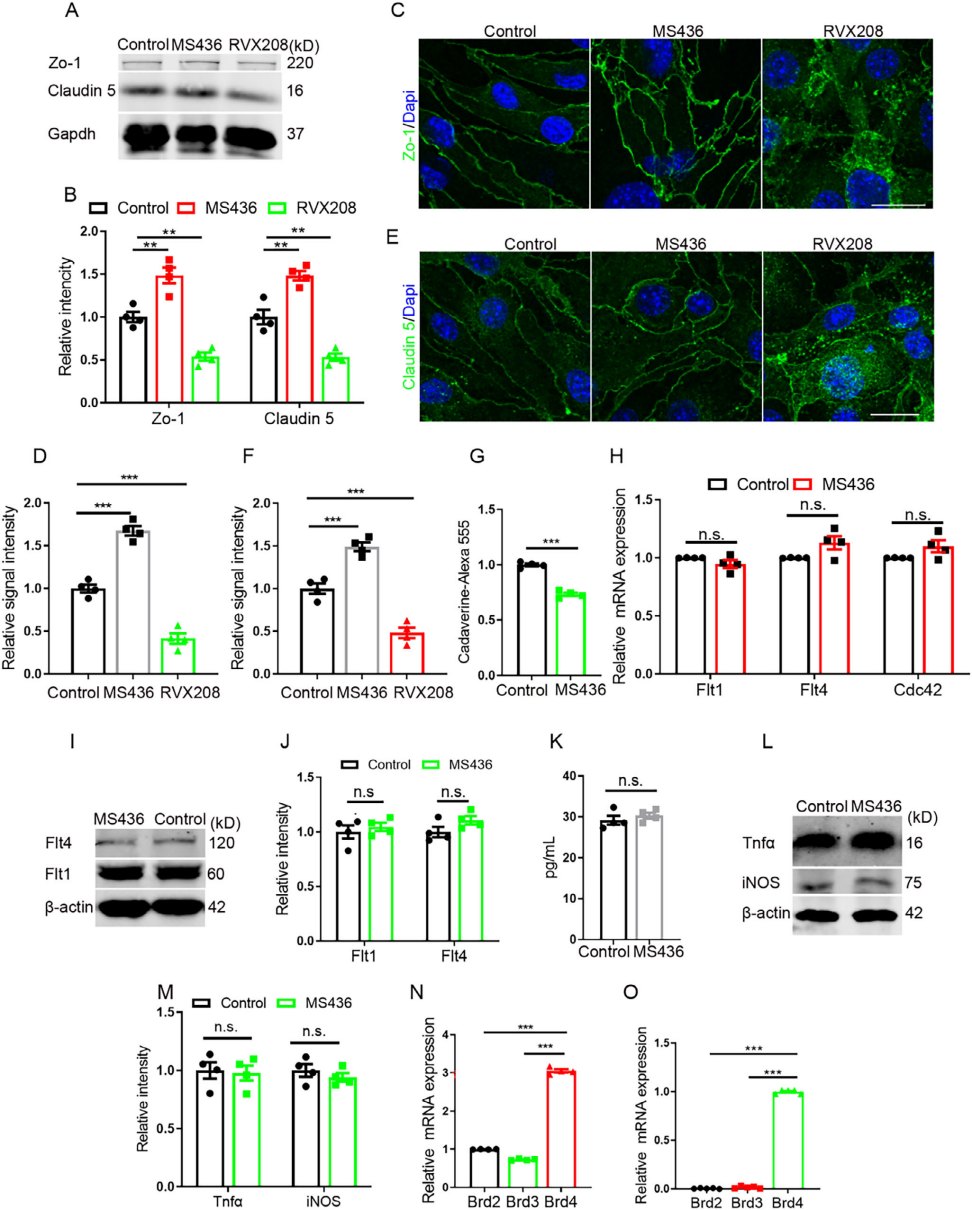
MS436 regulates the expression of TJ proteins. A) WB detection of the regulation of TJ by antagonists targeting BD domains(50 nM). B) Statistic analysis of relative intensity of TJ proteins in WB detection, n=4. C) Regulation of Zo‐1 by antagonists targeting BD domains (100 nM). Scale bar, 20 µm. D) Statistic analysis of relative signal intensity, n=4. E) Regulation of Claudin 5 by antagonists targeting BD domains (100 nM). Scale bar, 20 µm. F) Statistic analysis of relative signal intensity, n=4. G) Detection of the infiltration of Cadaverine 555 by trans‐well assays. n=4. H) Angiogenesis detection by RT‐PCR with bEnd.3 cells treated with MS436. n=4. I) WB detection of angiogenesis after MS436 treatment(100 nM). J) Statistic analysis of relative intensity of angiogenesis proteins in WB detection, n=4. K)Tnfα detection by ELISA after MS436 treatment, n=4. L) WB detection for inflammation factors with primary endothelilal cells. M) Statistic analysis of relative intensity of inflammation factors in WB detection, n=4. N) The level of BET family members detection by RT‐PCR with bEnd.3 cells, n=4. O) The level of BET family members detection by RT‐PCR with primary endothelilal cells at E14.5, n=4. Data were presented as mean ± SEM, one‐way ANOVA, Two‐tailed Student's *t*‐test, ns, no significant difference, *p < 0.05, **p < 0.01, ***p < 0.001.

To detect the effects of BET antagonists, we treated mouse bEnd.3 with two specific antagonists: MS436 (targeting BD1) and RVX208 (targeting BD2) respectively. Results showed that both antagonists affected the expression of tight junction (TJ) proteins, while they worked conversely. In detail, MS436 increased the expression level of TJ proteins of Zo‐1 and Claudin 5, while RVX208 decreased their expression levels (**Figure** **1**A,B). These findings were further confirmed by immunostaining of Zo‐1 and Claudin 5 (Figure 1C–F). In addition, consistent results were obtained using another set of antagonists of GSK778 (BD1) and GSK046 (BD2) (Figure S1A–D, Supporting Information). Since TJs serve as critical structural components of the BBB, their expression can partially reflect BBB functionality. Given the upregulation role of MS436 on the expression of TJ proteins, we focused our subsequent research on this compound. To further evaluate the biological significance of these findings, we established an in vitro BBB model using bEnd.3 cells and performed transwell permeability assays. The results demonstrated that MS436 treatment effectively reduced the paracellular permeability, as evidenced by decreased infiltration of Cadaverine 555 (Figure 1G). However, we did not observe its effect on angiogenesis (Figure 1H–J). Given the well‐documented anti‐inflammatory properties of BD antagonists, we sought to investigate whether inflammatory signaling pathways might mediate the observed enhancement of endothelial tightness following MS436 treatment. As a result, it demonstrated that MS436 treatment did not significantly alter inflammation‐related signaling pathways (Figure 1K–M).

**Figure 2:**
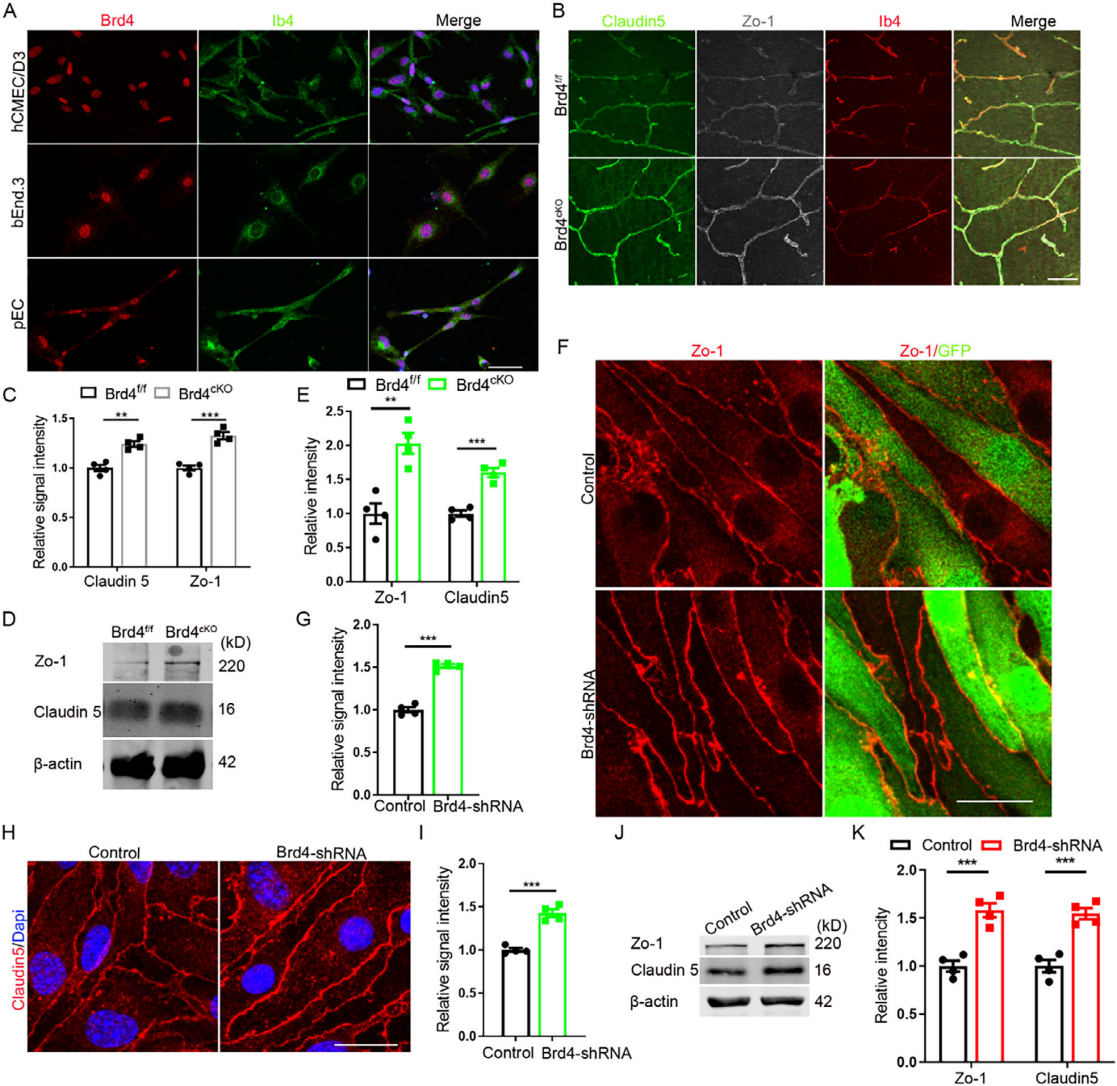
Brd4 regulates the expression of TJ proteins. A) Immunofluorescent staining for Brd4 with hCMEC/D3, bEnd.3 and pEC. Scale bar, 50 µm. B) Immunofluorescent staining for Claudin 5 and Zo‐1 with mice cerebral cortex at P1. Scale bar, 50 µm. C) Statistic analysis of relative signal intensity of TJ proteins, n=4. D) WB detection for TJ proteins with endothelilal cells taken from *Brd4^cKO^* cerebral cortex. E) Statistic analysis of relative intensity of TJ proteins in WB detection, n=4. F) Immunofluorescent staining for Zo‐1 with bEnd.3 lacking Brd4. Scale bar, 20 µm. G) Statistic analysis of relative signal intensity of Zo‐1, n=4. H) Immunofluorescent staining for Claudin 5 with bEnd.3 lacking Brd4 (acetone fixed). Scale bar, 20 µm. I) Statistic analysis of relative signal intensity of Claudin 5, n=4. J) WB detection for TJ proteins with endothelilal cells lacking Brd4. K) Statistic analysis of relative intensity of TJ proteins in WB detection, n=4. Data were presented as mean ± SEM, one‐way ANOVA, Two‐tailed Student's *t*‐test, ns, no significant difference, *p < 0.05, **p < 0.01, ***p < 0.001.

Because BET family has 4 members: Brd2, Brd3, Brd4, and BrdT, we next aimed to identify the specific downstream effector responsible for MS436's effects on TJ permeability regulation. Since the expression of BrdT predominantly restricted to testicular tissue,^[^ ^12^ ^]^ we focused our analysis on the remaining three members. Loss‐of‐function assays showed that both Brd2 and Brd4 changed the level of Zo‐1 and Claudin 5, while Brd3 changed the level of Claudin 5 only (Figure S1E,F,J,K, Supporting Information; **Figure** **2**J,K). RT‐PCR revealed that Brd4 exhibited the highest expression level among these BET family members (Figure 1N). To further explore the potential involvement of BET proteins in BBB development, we isolated brain vascular endothelial cells during the critical period of TJ formation at embryonic day 14.5 (E14.5) and performed RT‐PCR analysis. The results showed a striking expression pattern, with Brd4's levels exceeding those of Brd2 and Brd3 by more than 30‐fold (Figure 1O). This remarkable expression profile led us to concentrate our subsequent investigations on Brd4 as the primary candidate mediating MS436's effects on the functional regulation of BBB.

It is well established that both humans and mice possess two Brd4 isoforms.^[^ ^25^, ^26^ ^]^ To determine which isoform of Brd4 is functionally relevant in this context, we next conducted knockdown experiments. Our results demonstrated that the knockdown of its long isoform replicated the effects observed with MS436 treatment, whereas the knockdown of the short isoform produced the opposite outcome (Figure S1G,H, Supporting Information), aligns with findings from other studies.^[^ ^27^, ^28^ ^]^ Additionally, we observed a significant disparity in expression levels, with the long isoform being expressed at more than 20‐fold higher levels compared to the short isoform (Figure S1I, Supporting Information). Based on these findings, we focused our subsequent investigations on the long isoform.

### 2.2 Brd4 Affects the Formation of Tight Junction

Brd4 functions critically both in the initiation^[^ ^29^ ^]^ and elongation^[^ ^30^ ^]^ of transcription. Antagonists targeting Brd4 are promising in the treatment of brain diseases such brain inflammation diseases,^[^ ^22^, ^31^ ^]^ autism^[^ ^32^ ^]^ and so on. While such studies related brain development and brain diseases are scarce, more exquisite assays are needed to distinguish their special function during brain development and the progression of brain diseases. To consolidate the expression of Brd4 in developing brain, we conducted Brd4 staining in developing brain. The results showed that compared to other cell types, Brd4 expressed highest in endothelial cells (Figure S2A, Supporting Information). This distinct expression profile further supports the potential role of Brd4 in cerebrovascular development and BBB formation. In vitro studies consolidated the expression of Brd4 in human cerebral microvascular endothelial cell Line/D3 (hCMEC/D3), mouse bEnd.3 and mouse primary endothelial cells (pEC) (Figure 2A). To further investigate the role of endothelial Brd4, we deleted endothelial *Brd4* by crossing Brd4‐flox mice with Tie2‐cre mice (*Brd4^cKO^*) (Figure S2B, Supporting Information). The deletion of Brd4 was timed to precede BBB formation, as Tie2 expression initiates around embryonic day 7 (E7) in mice. The successful and specific knockout of Brd4 in the endothelial cell lineage was subsequently validated by immunostaining (Figure S2C, Supporting Information). This was confirmed by WB assays as well (Figure S2D,E, Supporting Information). Compared with their littermates, *Brd4^cKO^* mice show similar angiogenesis (Figure S2F–I, Supporting Information) and pericytes attachment (Figure S2J,K, Supporting Information). Flow cytometer sorting and RT‐PCR revealed that Brd4 expressed highest at E14, which was consistent with the peak stage of TJ formation (Figure S2L, Supporting Information). This implied that Brd4 might participate in the regulation of TJ protein expression. To confirm this, we conducted TJ protein staining, immunostaining of Claudin 5 and Zo‐1 indicated that compared with their littermates, *Brd4^cKO^* mice had higher level of TJ proteins (Figure 2B,C), and WB analysis showed consistent findings (Figure 2D,E). Meanwhile, we knocked down *Brd4* in bEnd.3 cells and conducted staining of TJ proteins, which consistently mimicked the in vivo results of immunostaining (Figure 2F–I) and WB assays (Figure 2J,K). Together, above results demonstrated that Brd4 is a negative regulator of TJ protein expression.

### 2.3 Rnf43 is the Main Downstream Target of Brd4

**Figure 3:**
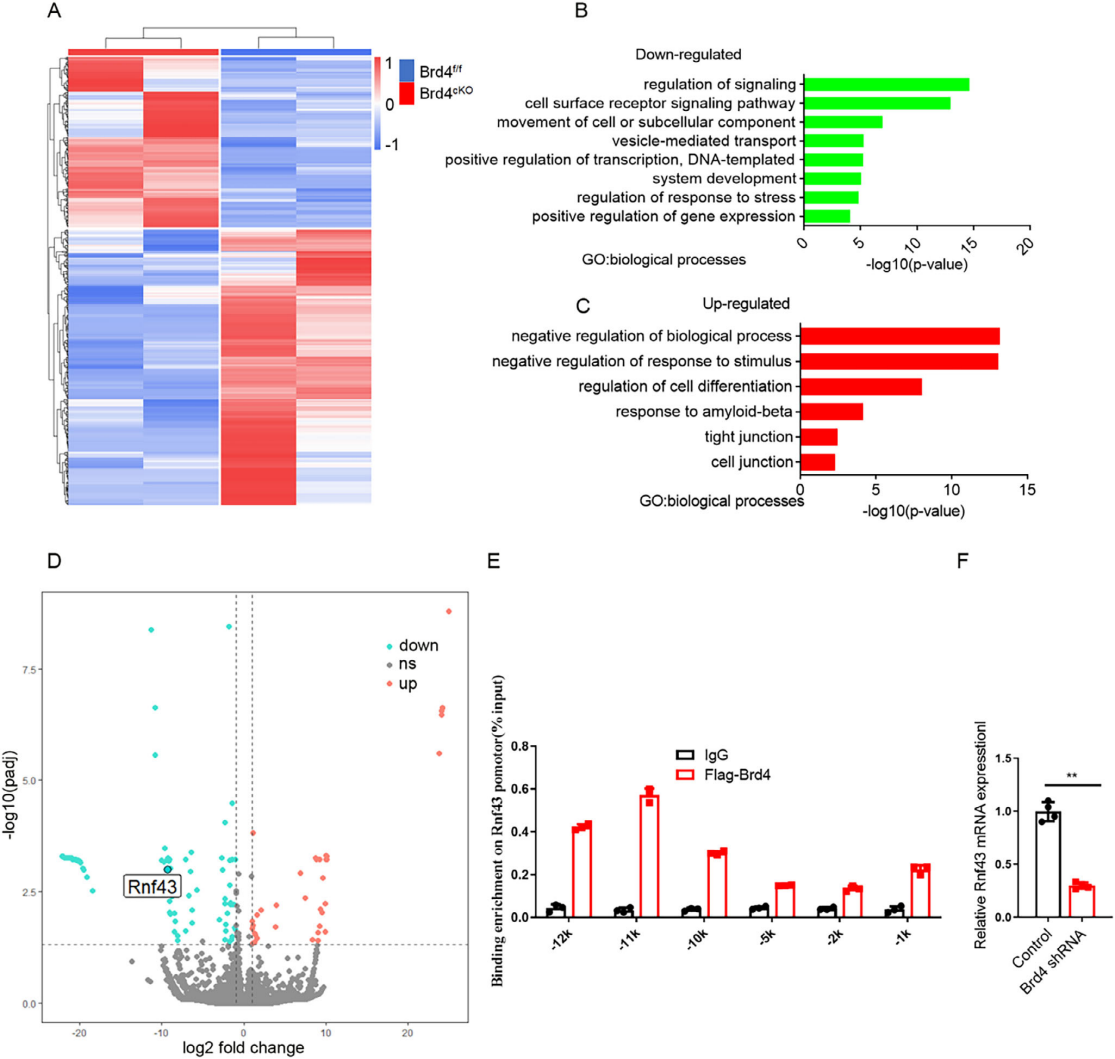
RNA‐seq analysis of mice brain vascular endothelial cells. A) Heap map of RNA sequencing data of brain vascular endothelial cells from E14.5 mice. B,C) Down‐regulated and up‐regulated signaling pathways by Gene ontology analysis. D) Distribution of down‐regulated and up‐regulated genes showed by Volcano plots. E) Detection of binding potential of Brd4 on the promotor of *Rnf43*. n=3. F) The level of Rnf43 detection by RT‐PCR with bEnd.3 cells after *Brd4* knock‐down. n=4. Data were presented as mean ± SEM, one‐way ANOVA, Two‐tailed Student's *t*‐test, ns, no significant difference, *p < 0.05, **p < 0.01, ***p < 0.001.

To unveil the underlying regulating mechanism of Brd4 on TJ formation, we sorted endothelial cell from mice cerebral cortex and conducted RNA sequencing (RNA‐Seq). Heat map analysis indicated that absent of Brd4 tremendously altered gene expression levels (**Figure** **3**A). Gene ontology analysis showed the down‐regulated signalilng pathways were mainly focused on gene transcription, vesicle mediated transport and movement of cell or subcellular components (Figure 3B), and the up‐regulated signaling pathways were mainly related to cell junction, TJ (Figure 3C), which were partially consistent with the observed phenomenon of TJ enhancement.

Next, we focused on a significantly downregulated gene of Rnf43 (Ring Finger Protein 43) as the main downstream target of Brd4 (Figure 3D). Rnf43, an E3 ubiquitin‐protein ligase, is a well‐established negative regulator of both canonical and non‐canonical Wnt signaling pathways.^[^ ^33^, ^34^, ^35^ ^]^ Although previous studies have primarily focused on its roles in tumor regulation^[^ ^12^, ^36^ ^]^ and stem cells self‐renewal,^[^ ^37^, ^38^ ^]^ its function in vascular biology remains largely unexplored. To explore whether Brd4 modulates TJ integrity through Rnf43, we first performed ChIP analysis and the results confirmed that Brd4 could bind to the distal promotor of *Rnf43* (Figure 3E). Meanwhile, RT‐PCR with bEnd.3 extraction showed that *Brd4* knockdown significantly decreased the expression level of *Rnf43* (Figure 3F).

**Figure 4:**
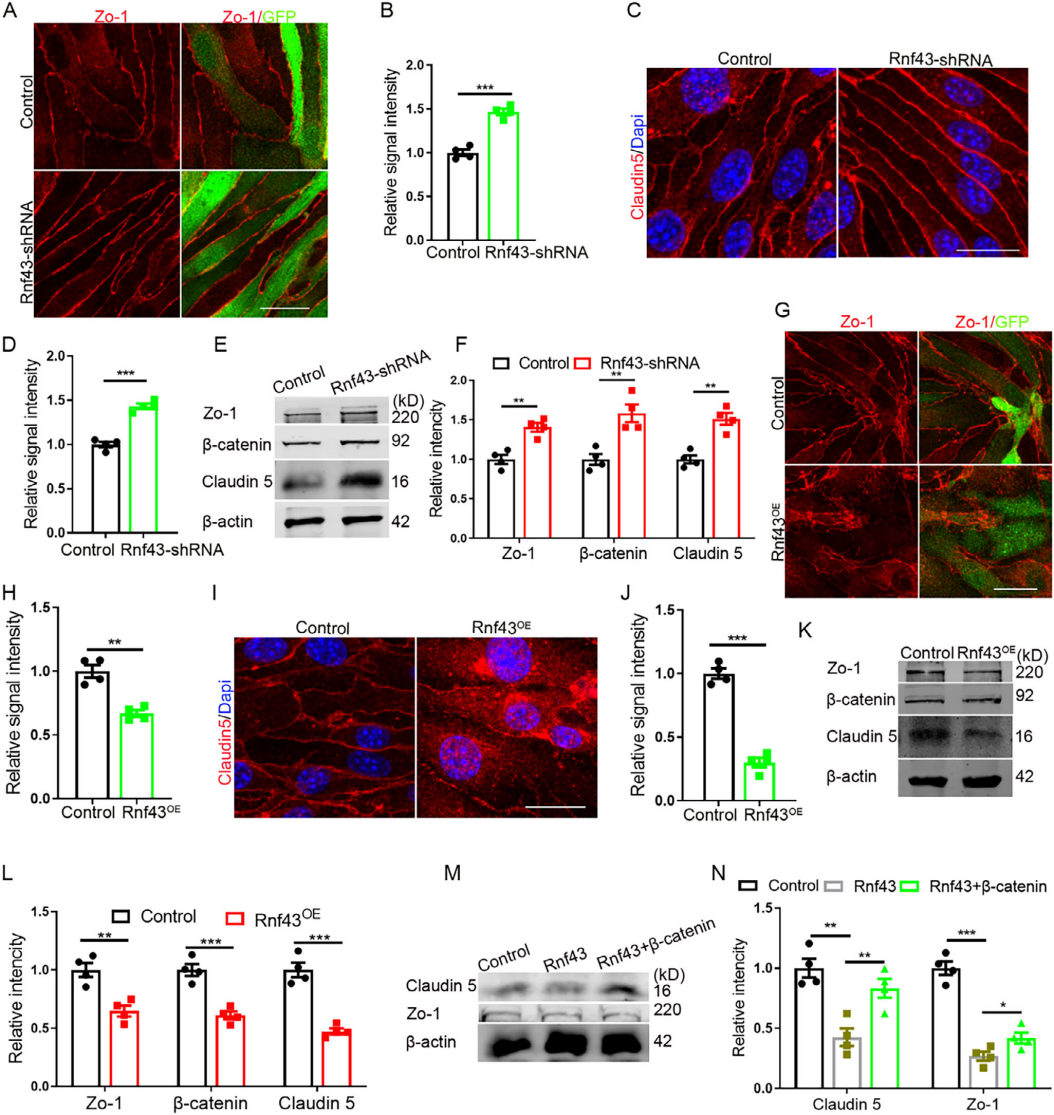
Rnf43 negatively regulates the expression of TJ proteins. A) Immunofluorescent staining for Zo‐1 with bEnd.3 lacking Rnf43. Scale bar, 20 µm. B) Statistic analysis of relative intensity of Zo‐1, n=4. C) Immunofluorescent staining for Claudin 5 with bEnd.3 lacking Rnf43 (acetone fixed). Scale bar, 20 µm. D) Statistic analysis of relative intensity of Claudin 5, n=4. E) WB detection for TJ proteins and β‐catenin with bEnd.3 lacking Rnf43. F) Statistic analysis of relative intensity of TJ proteins and β‐catenin in WB detection, n=4. G) Immunofluorescent staining for Zo‐1 with bEnd.3 overexpressing Rnf43. Scale bar, 50 µm. H) Statistic analysis of relative intensity of Zo‐1, n=4. I) Immunofluorescent staining for Claudin 5 with bEnd.3 overexpressing Rnf43 (acetone fixed). Scale bar, 20 µm. J) Statistic analysis of relative intensity of Claudin 5, n=4. K) WB detection for TJ proteins and β‐catenin with bEnd.3 overexpressing Rnf43. L) Statistic analysis of relative intensity of TJ and β‐catenin in WB detection, n=4. M) WB detection for TJ proteins with bEnd.3 overexpressing Rnf43 and β‐catenin. N) Statistic analysis of relative intensity of TJ proteins in WB detection, n=4. Data were presented as mean ± SEM, one‐way ANOVA, Two‐tailed Student's *t*‐test, ns, no significant difference, *p < 0.05, **p < 0.01, ***p < 0.001.

Next, by performing loss‐of‐function experiments in bEnd.3 cells, it revealed that *Rnf43* knockdown resulted in a significant upregulation of TJ proteins (**Figure** **4**A–D), a phenotype that closely mirrored the effects observed in Brd4‐deficient cells. Western blot analysis confirmed these findings at the protein level (Figure 4E,F). Further mechanistic studies revealed that *Rnf43* depletion led to the accumulation of β‐catenin, suggesting that Rnf43 might regulate TJ dynamics through the canonical Wnt/β‐catenin signaling pathway. This hypothesis was supported by reciprocal gain‐of‐function experiments, where Rnf43 overexpression conversely reduced TJ protein levels (Figure 4G–L). Notably, β‐catenin overexpression could partially rescue the Rnf43‐mediated downregulation of TJ (Figure 4M,N), while knockdown of *β‐catenin* attenuated the TJ structure‐enhancing effects caused by *Brd4* depletion (Figure S3A, B, Supporting Information). These results establish a functional link between Brd4, Rnf43, and Wnt/β‐catenin signaling on the regulation of endothelial TJ integrity.

### 2.4 Fzd4 is the Downstream Target of Rnf43

**Figure 5:**
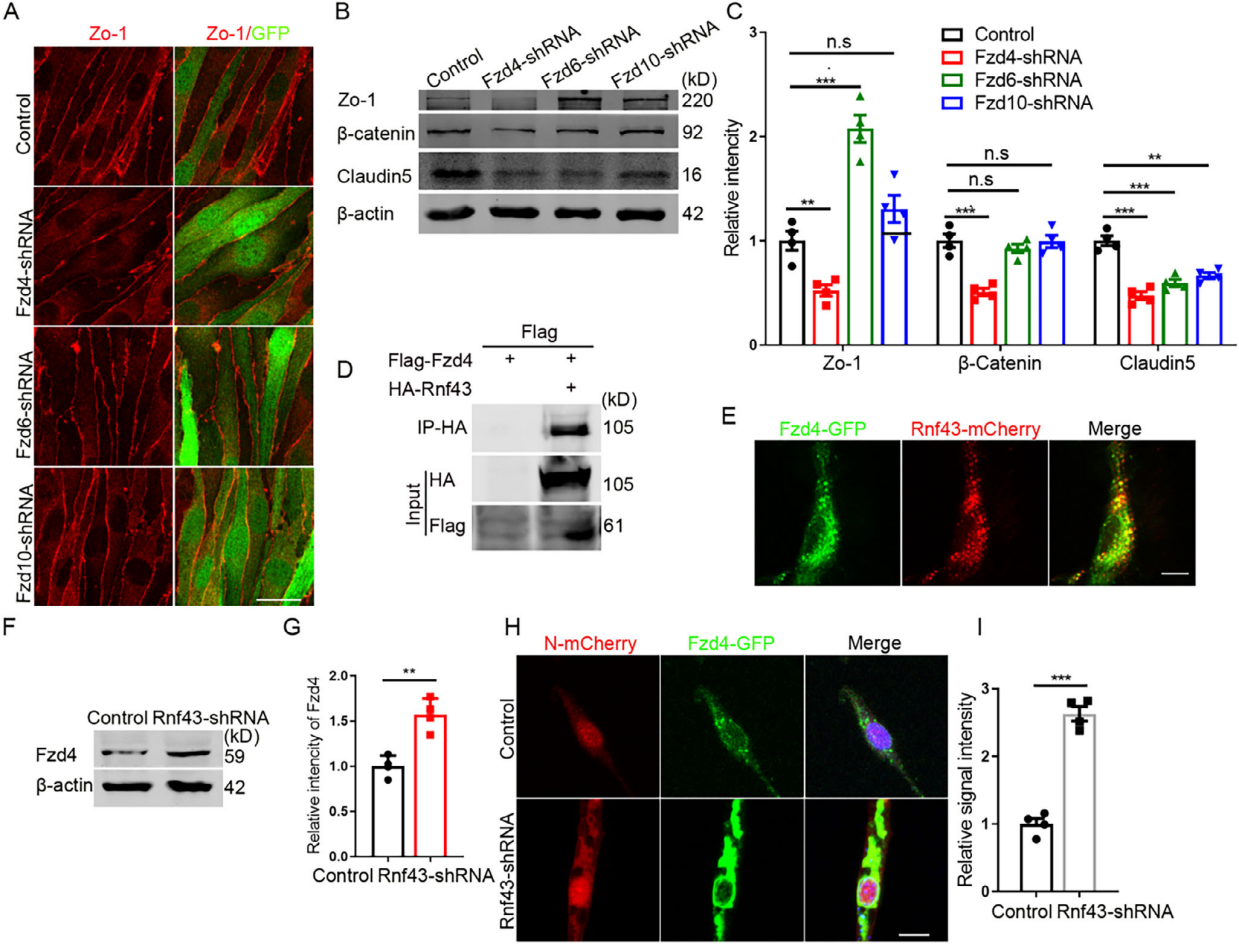
Rnf43 regulates the level of TJ through Fzd4. A) Immunofluorescent staining for Zo‐1 with bEnd.3 lacking Fzd4, Fzd6 or Fzd10. Scale bar, 50 µm. B) WB detection for TJ proteins and β‐catenin with bEnd.3 lacking Fzd4, Fzd6 or Fzd10. C) Statistic analysis of relative intensity of TJ proteins and β‐catenin in WB detection, n=4. D) Co‐IP detection of Rnf43 and Fzd4. E) Co‐location detection of Rnf43 and Fzd4. Scale bar, 10 µm. F) WB detection for Fzd4 after *Rnf43* knockdown. G) Statistic analysis of relative intensity of Fzd4 in WB detection, n=4. H) Detection of Fzd4 after *Rnf43* knockdown. Scale bar, 10 µm. I) Statistic analysis of relative signal intensity of Fzd4, n=4. Data were presented as mean ± SEM, one‐way ANOVA, Two‐tailed Student's *t*‐test, ns, no significant difference, *p < 0.05, **p < 0.01, ***p < 0.001.

It has been reported that Rnf43 could negatively regulates β‐catenin through Frizzled (Fzd) family members, while which member linked it and β‐catenin in endothelial cells is still unknown. By checking the brain sequencing data, we found Fzd4, Fzd6, Fzd10 expressed highly in developing brain. We therefore knocked them known in bEnd.3 cells respectively and conducted the detection of Zo‐1. The results showed that the knockdown of *Fzd4* efficiently decreased the expression level of Zo‐1 (**Figure** **5**A). WB analyses showed that the knockdown of *Fzd4* considerably reduced the level of both TJ proteins and β‐catenin (Figure 5B,C). These data suggested that Rnf43 might regulate the expression level of β‐catenin through Fzd4. Therefore, we next aimed to explore evidences supporting the regulation of Rnf43 on Fzd4. By performing Co‐IP and co‐staining assays, we demonstrated a direct reaction and interaction between Rnf43 and Fzd4 (Figure 5D,E). Moreover, it was found that Rnf43 significantly increased the ubiquitylation of Fzd4 (Figure S4A, Supporting Information). Meanwhile, loss of Rnf43 increased the expression level of Fzd4 (Figure 5F–I), while knockdown of *Fzd4* could reversely restore the downregulated expression of TJ proteins induced by *Rnf43* knockdown (Figure S4B,C,D,E, Supporting Information). Above results revealed that Rnf43 could specifically target Fzd4 via ubiquitin‐proteasome degradation, thereby suppressing β‐catenin signaling and destabilizing TJ proteins, unveiling the Rnf43/Fzd4 axis as a core regulatory mechanism for BBB integrity

### 2.5 The BD1 and BD2 of Brd4 Function on the Regulation of TJ Proteins Conversely

**Figure 6:**
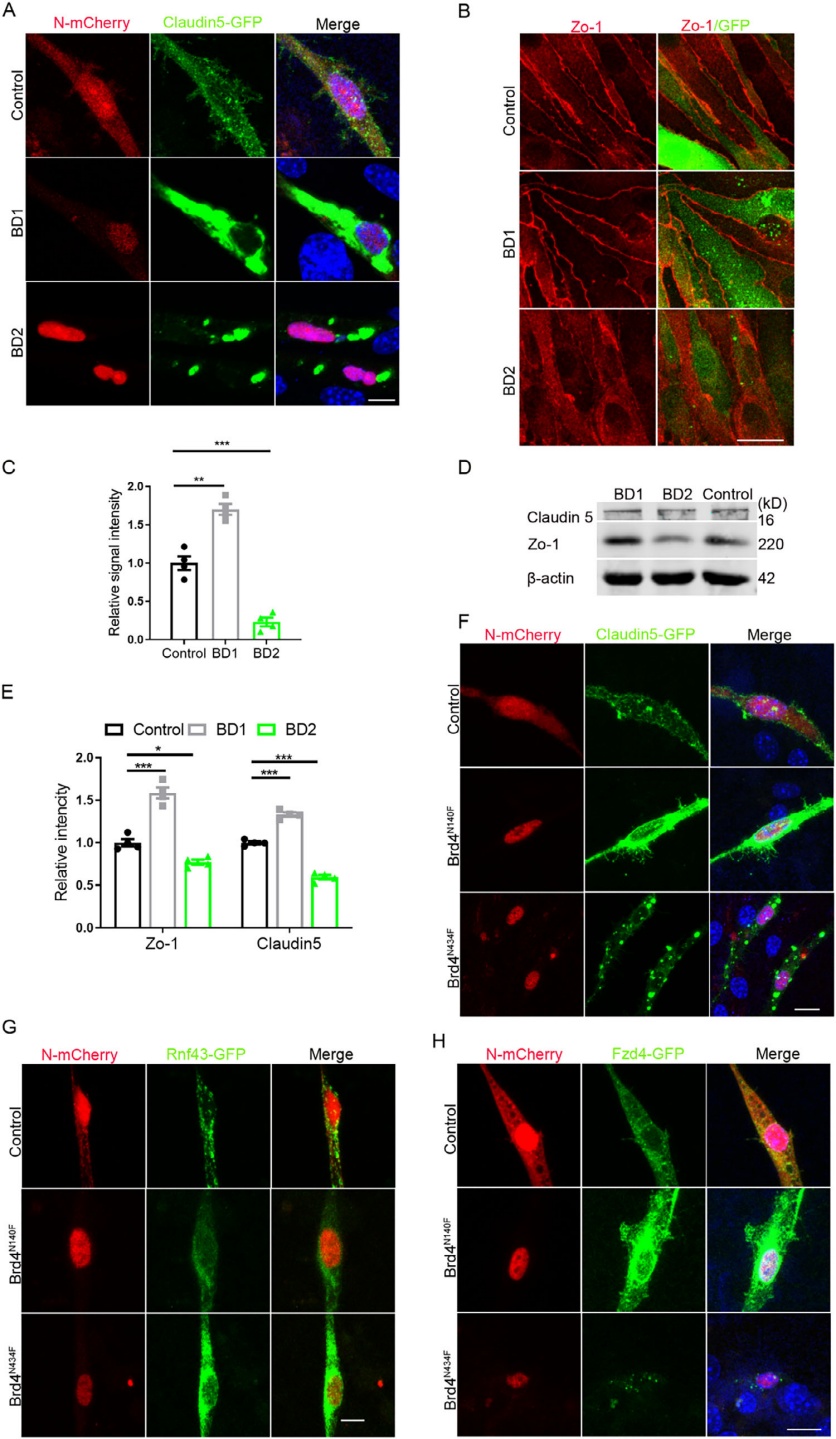
Brd4 regulates the expression of TJ proteins depends on its BD1 domain. A) Regulation of Claudin 5 by BD domains. Scale bar, 10 µm. B) Immunofluorescent staining for Zo‐1 with bEnd.3 overexpressing BD1 and BD2. Scale bar, 20 µm. C) Statistic analysis of relative intensity of Zo‐1, n=4. D) WB detection for TJ proteins with bEnd.3 overexpressing BD1 or BD2. E) Statistic analysis of relative intensity of TJ proteins in WB detection, n=4. F) Regulation of Claudin 5 by Brd4 mutants. Scale bar, 10 µm. G) Detection of the regulation of *Brd4* mutants on the expression of Rnf43. H) Detection of Fzd4 after mutation of *Brd4*. Scale bar, 10 µm. Data were presented as mean ± SEM, one‐way ANOVA, Two‐tailed Student's *t*‐test, ns, no significant difference, *p < 0.05, **p < 0.01, ***p < 0.001.

Although above results have established a correlation between BD1 antagonism and TJ protein expression, the specific contribution of Brd4 domains to this process remains to be elucidated. To directly investigate the functional relationship between Brd4's BD domains and TJ integrity, we employed a domain‐specific overexpression strategy. By separately overexpressing the BD1 and BD2 domains to competitively bind their respective downstream targets, their individual roles on the regulation of TJ protein expression are thereby dissected. Intriguingly, the results demonstrated distinct domain‐specific effects: overexpression of the BD1 domain phenocopied the TJ‐enhancing effects observed in *Brd4* knockdown conditions, suggesting a dominant‐negative effect on endogenous Brd4 function. In contrast, BD2 domain overexpression led to increased degradation of TJ, indicating an opposing regulatory role for this domain (**Figure** **6**A–C). These findings provide direct evidence supporting that the BD1 domain of Brd4 plays a pivotal role in maintaining TJ integrity, while the BD2 domain might function in a counter‐regulatory capacity. Western blot analysis corroborated these findings (Figure 6D,E).

To further validate the domain‐specific effects, we generated targeted mutations at the binding sites of individual BD domains. Mutation of the BD1 binding site (N140F) significantly increased the expression level of Claudin 5, while mutation of the BD2 binding site (N434F) significantly decreased the protein level of Claudin 5 (Figure 6F). Mutation of the BD1 binding site also significantly attenuated Brd4‐mediated transcriptional activation of *Rnf43*, while mutation of the BD2 binding site showed no appreciable effect on *Rnf43* transcription (Figure 6G). Interestingly, the mutation of BD1 binding site resulted in an upregulated Fzd4 expression, phenocopying the effects observed in *Rnf43* knockdown conditions (Figure 6H). These results suggest a potential mechanistic link between BD1‐mediated TJ regulation and Wnt signaling pathway modulation through Rnf43/Fzd4 axis.

### 2.6 MS436 Prevents BBB Disruption in Stroke

**Figure 7:**
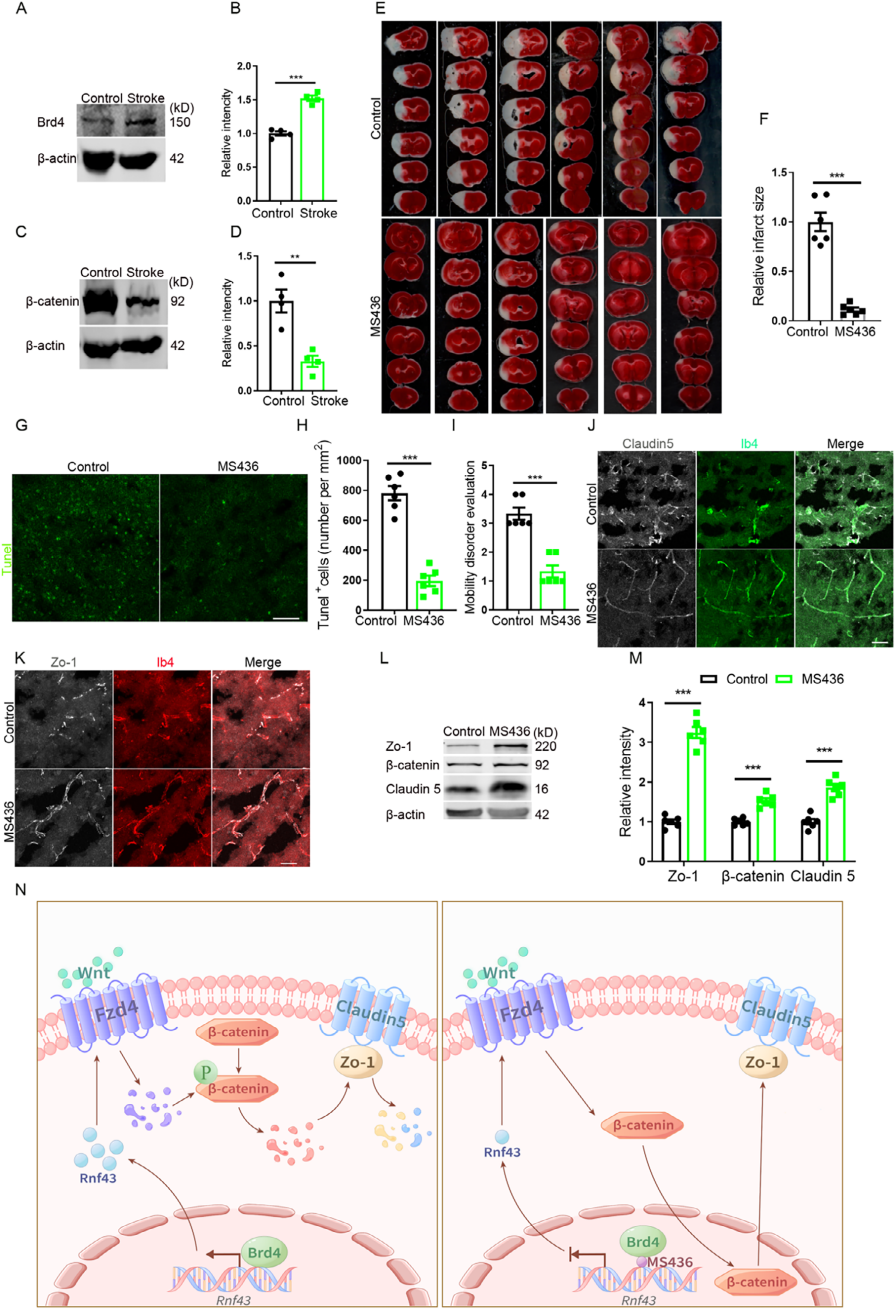
MS436 alleviates BBB injury induced by stroke. A) WB detection for Brd4 with mice cerebral cotex after stroke. B) Statistic analysis of relative intensity of Brd4 in WB detection, n=4. C) WB detection for β‐catenin with mice cerebral cotex after stroke. D) Statistic analysis of relative intensity of β‐catenin in WB detection, n=4. E) Infarct size evaluation by TTC staining after stroke. F) Relative infarct size evaluation, n=6. G) Tunel staining with mice brain after stroke. Scale bar, 50 µm. H) Statistic analysis of Tunel positive cells, n=6. I) Mobility disorder evaluation, n=6. J) Staining of Claudin 5 in mice brain after stroke. Scale bar, 20 µm. K) Staining of Zo‐1 in mice brain after stroke. Scale bar, 20 µm. L) WB detection for TJ and β‐catenin with mice cerebral cortex after stroke. M) Statistic analysis of relative intensity of Clauidn 5 and β‐catenin in WB detection, n=6. N) Schematic diagram of the mechanism MS436 inhibits Brd4's function and suppresses Rnf43‐mediated β‐catenin destabilization and TJ protein degradation, therefore protects BBB integrity. Data were presented as mean ± SEM, one‐way ANOVA, Two‐tailed Student's *t*‐test, ns, no significant difference, *p < 0.05, **p < 0.01, ***p < 0.001.

As a debilitating cerebrovascular pathology, stroke remains a leading global health challenge marked by substantial mortality rates and persistent neurological impairments. A critical pathophysiological hallmark of cerebral ischemia in stroke involves severe BBB disruption. Given our previous findings on the critical role of Brd4 in BBB regulation, we hypothesized that Brd4 might play a previously unrecognized role in the pathophysiological processes of stroke. To test this hypothesis, we employed the ischemic stroke research of middle cerebral artery occlusion (MCAO) mouse model. Intriguingly, our data revealed a significant upregulation of Brd4 expression (**Figure** **7**A,B) and a significant downregulation of β‐catenin expression (Figure 7C,D) in the ischemic brain following MCAO induction, suggesting their potential involvement in stroke‐related cellular responses.

Next, to investigate the therapeutic potential of MS436 in cerebral ischemic mice, we administered the compound immediately upon reperfused MCAO mice from anesthesia and collected brain tissues 12 h post‐treatment for comprehensive analysis. We conducted TTC (2,3,5‐Triphenyltetrazolium Chloride) staining, a reliable technique for quantifying cerebral infarction volume in experimental stroke research.^[^ ^39^ ^]^ The staining results showed that MS436‐treated mice displayed a remarkable reduction in cerebral infarct volume compared with controls (Figure 7E,F). These in vivo evidences demonstrate notable neuroprotective effects of MS436 in cerebral vasculatures suffering ischemic injuries. This neuroprotective effect was further corroborated by TUNEL staining, which showed significantly fewer apoptotic cells in the treatment group (Figure 7G,H). Meanwhile, behavioral assessments through standardized mobility test were also performed and the results demonstrated that MS436‐treated mice exhibited substantially improved neurological function and reduced motor impairments (Figure 7I). Given the established role of MS436 in preserving TJ integrity in above in vitro studies, we hypothesized that its neuroprotective effects might be mediated through BBB stabilization. Hence, we performed immunohistochemical analysis of TJ proteins to support this hypothesis. The results showed enhanced TJ protein expression and integrity in MS436‐treated mice (Figure 7J,K). WB analysis further revealed that MCAO mice treated with MS436 expressed higher level of both TJ and β‐catenin (Figure 7L,M). Evens blue staining showed that MS436 greatly reduced the BBB leaky (Figure S5A,B, Supporting Information). It is of particular interest that we found comparable serum TNF‐α level in both groups, which indicates that the mechanism of action is not mediated by the inhibition of systemic inflammation (Figure S5C, Supporting Information). These findings collectively suggest that MS436 exerts its neuroprotective effects, at least in part, through preservation of BBB integrity following ischemic insult.

In summary, our study reveals that MS436 protects BBB integrity by selectively inhibiting Brd4's BD1 domain, which suppresses Rnf43‐mediated β‐catenin destabilization and TJ protein degradation, therefore attenuating BBB leakage and improved neurological outcomes (Figure 7N). These findings redefine Brd4 BD1 inhibition as a novel therapeutic strategy for neurovascular disorders and underscore the critical role of domain‐specific BET targeting in preserving BBB function.

## 3 Discussion

Substantial evidence indicates that BBB dysfunction is a common pathological feature across various neurodegenerative disorders, including PD,^[^ ^40^ ^]^ AD,^[^ ^2^, ^41^ ^]^ MS,^[^ ^42^, ^43^ ^]^ and Huntington's disease,^[^ ^44^ ^]^ as well as acute neurological conditions such as stroke.^[^ ^45^, ^46^ ^]^ While these findings strongly suggest an intricate relationship between BBB dysfunction and neurodegenerative disease pathogenesis, the precise causal mechanisms remain to be fully elucidated. Meanwhile, the therapeutic potential of BBB‐targeting interventions for neurodegenerative diseases warrants further investigation through rigorous preclinical and clinical studies. In this study, we have identified MS436, a known antitumor agent, as a potent protector of BBB integrity. Our mechanistic investigations reveal that Brd4 serves as the primary downstream effector of MS436, mediating its protective effects through modulation of the Wnt signaling pathway, which subsequently influences TJ protein expression (Figure 7M).

Although BET antagonists have been explored for treating BBB‐related disorders—including neurodegenerative and acute cerebrovascular diseases—their therapeutic effects are primarily attributed to anti‐inflammatory mechanisms. The specific roles of BDs in cerebrovascular pathophysiology remain unclear, and domain‐selective BD antagonists have not been systematically evaluated for these conditions. Notably, we found that the BD1‐specific antagonist MS436, unlike broad‐spectrum BD antagonists, exerts significant benefits in stroke models by attenuating BBB disruption and improving functional outcomes through an inflammation‐independent mechanism. This represents a novel therapeutic strategy that directly targets BBB integrity for treating BBB‐associated pathologies. Our findings demonstrate that BBB‐protective agents might serve as promising first‐line interventions for acute vascular events, particularly stroke. Importantly, the data suggest that BBB disruption is not merely an epiphenomenon but rather plays a causative role in disease pathogenesis. These results also highlight the potential therapeutic value of BBB protection in various neurodegenerative disorders, including AD, PD, and MS.

Based on our mechanistic insights, we propose that pan‐Brd4 antagonists targeting both bromodomains may not be optimal for BBB‐related disorders. Instead, our findings emphasize the need for further research to elucidate the distinct functions of individual bromodomains across different disease states and progression stages, which could inform the development of more targeted therapeutic strategies.

While our study provides valuable insights, several limitations should be acknowledged. First, although we have partially addressed the involvement of inflammatory signaling pathways, the potential effects of MS436 on inflammation in MCAO models remain to be fully elucidated. More sophisticated experimental approaches are required to dissect the specific contribution of BBB protection from potential anti‐inflammatory effects. Second, while we have demonstrated that the two bromodomains of BET proteins exert opposing effects on downstream events, our mechanistic investigation has primarily focused on one domain. The molecular mechanisms underlying the function of the other bromodomain remain to be elucidated. This limitation is further compounded by the current lack of highly specific antagonists targeting individual bromodomains. The development of more selective pharmacological tools will be crucial for minimizing off‐target effects and validating our findings. Third, we observed an apparent functional antagonism between the Brd4 isoforms, with the long form being overwhelmingly more abundant. How the minor short isoform exerts significant biological influence under these conditions is a key question. The finding that the short isoform modulates the long isoform's phase separation leads us to a central hypothesis: their functions are interdependent.^[^ ^25^ ^]^ A critical limitation, however, is that these observations are based on mRNA quantification; validating this model at the protein level necessitates the development of antibodies that can discriminate between the two isoforms. Fourth, without pharmacokinetics, BBB penetration, or toxicity assessment, MS436 employment is far less in the treatment of stroke in human. From a broader perspective, the therapeutic strategy of prioritizing BBB protection as a primary emergency intervention would benefit from comparative studies with other established BBB protectants in stroke models. Such investigations would help validate the clinical relevance of our approach and provide a more comprehensive understanding of BBB‐targeted therapies.

## 4 Experimental Section

### Mice

The *Tie2‐Cre* (*TEK‐Cre*) mice were obtained from the Shanghai Model Organism. Mice were raised under the conditions of 12 h (h) light and 12 h darkness. All of the mice experimental procedures were approved by the Animal Committee of Institute of Zoology, Chinese Academy of Sciences and the Animal Committee of Guangdong Medical University (No. GDY2402529).

### Generation of *Brd4^cKO^* Mice

The *Brd4^flox/flox^* mice were generated according to the procedures as previously described.^[^ ^47^ ^]^ *Brd4^cKO^* mice were obtained by crossing *Brd4^flox/flox^* mice with *Tie2‐Cre* mice, genotyping PCR primers were shown in Table S1 (Supporting Information). Tie2 expresses begin from about E7 in mice,^[^ ^48^, ^49^ ^]^ and Brd4 was KO from about E7.

### Cell Culture

The human embryonic kidney cells (HEK293FT) (Hunan Fenghui Biotechnology Co., Ltd, CL0004), the human brain endothelial cell line (hCMEC/D3) (Millipore, SCC066) and brain‐derived endothelial cells (bEnd.3) (ATCC, CRL‐2299) were from Dr. Jianwei Jiao's lab in the Key Laboratory of Organ Regeneration and Reconstruction, Chinese Academy of Science, Beijing. The cell lines were contamination free. HEK293T and bEnd.3 were cultured in high glucose DMEM medium supplemented with 10% fetal bovine serum and 1% penicillin/streptomycin. hCMEC/D3 were cultured with ECM. All cells were cultured at 5% CO2 and 37 °C temperature. Genes overexpression and knockdown in bEnd.3 and pEC were conducted by lentivirus

### Lentivirus Production and Infection

Lentivirus plasmid and viral plasmid backbone were transfected into HEK293FT cells by GenEscortII (Wisegen Wis 2400) for 4 h. DMEM supernatant containing virus were collected at 24, 48, and 72 h. For infection, cells were cultured with a mixture with a half DMEM and a half virus in the presence of 4 µg mL^−1^ Polybrene(Sigma TR‐1003‐G) for 10 h.

### Isolation and Culture of Primary ECs

Mice brain at around E14 was dissected under stereomicroscopy, meninges, olfactory bulb and brainstem were removed. Cerebral cortex was digested with 20 U mg^−1^ papain (Worthington, Ls003119) for 5 min at 37 °C. After filtered with 70 µm filter, cell suspension was counted. 0.3 million or 3 million cells per well were plated onto 24 well or 6 well plates (coated with Rat Collagen Type I, Sigma C3867‐1VL) with ECM (Sciencell 1001), cells were cultured at 5% CO2 and 37 °C temperature.

### RNA‐Sequencing Analysis

Mice brains of E14 were dissected under stereomicroscopy, meninges, olfactory bulb and brainstem were removed. Cerebral cortex was digested with 20 U mg^−1^ papain (Worthington, Ls003119) for 5 min at 37 °C. After filtered with 70 µm filter, cell suspension was counted. 10^7^ cells in 1 mL PBS were incubated with CD31‐FITC antibody (Biolegend 102 406) at 37 °C for 30 min and then were sorted by flow cytometry. Total RNA was subsequently extracted using the Tiangen RNAprep Pure Micro Kit (Catalog #DP420). Following quality control assessment, cDNA libraries were constructed and subjected to high‐throughput sequencing on the Illumina HiSEq 2500 platform.

### Immunostaining

The procedure was performed as previously described.^[^ ^50^ ^]^ Briefly, samples were fixed in 4% paraformaldehyde for 24–48 h, followed by dehydration in 30% sucrose for 24 h. After sectioning, samples were post‐fixed in 4% paraformaldehyde for 30 min and subsequently washed three times with PBS containing 0.1% Triton X‐100. Non‐specific binding was blocked with 5% bovine serum albumin for 1 h at room temperature. Samples were then incubated with primary antibody overnight at 4 °C. Following three washes, sections were incubated with appropriate secondary antibodies for 1 h at room temperature. For TUNEL staining, the Transgen Biotech TUNEL Kit was used according to the manufacturer's protocol: brain sections were incubated with a reaction mixture containing 50 µL 1× labeling solution and 2 µL TdT enzyme at 37 °C for 30 min. After final washes, immunostained slides were imaged using a Zeiss LSM 880 confocal microscope.

### Western Blotting and Co‐Immunoprecipitation

Protein extraction was performed using RIPA lysis buffer supplemented with 1% PMSF and 1% protease inhibitor cocktail. Cell lysates were sonicated and centrifuged at 12000 × *g* for 15 min at 4 °C, and the supernatants were collected. Protein concentration was determined using the BCA assay. Samples were denatured by boiling in Laemmli buffer for 7 min at 95 °C and subsequently separated by 10% or 12% SDS‐PAGE. Proteins were then transferred onto nitrocellulose membranes using a wet transfer system. Membranes were blocked with 5% BSA or non‐fat milk in PBST (PBS containing 0.05% Tween‐20) for 1 h at room temperature, followed by incubation with primary antibodies overnight at 4 °C. After three washes with PBST, membranes were incubated with appropriate HRP‐conjugated secondary antibodies for 1 h at room temperature. Following three additional washes, protein bands were visualized using enhanced chemiluminescence and quantified using Image Studio Ver 5.2 software. β‐actin or GAPDH served as loading controls.

For co‐immunoprecipitation experiments, protein lysates were incubated with anti‐HA‐tag (MBL) or anti‐Flag‐tag magnetic beads (MBL) overnight at 4 °C with gentle rotation. Beads were washed three times with ice‐cold lysis buffer, and bound proteins were eluted by boiling in 2 x Laemmli buffer for subsequent western blot analysis.

### Real‐Time PCR (RT‐PCR)

Total RNA was extracted by TRIzol (Invitrogen, 15 596) method and reverse transcribed into cDNA by FastQuant RT Kit (Tiangen). SYBR qPCR master mix (Tiangen) was used for RT‐PCR by ABI7500 real‐time PCR system (Applied Biosystems). Data normalization was performed based on 𝛽‐actin.

### MCAO (Middle Cerebral Artery Occlusion)

C57 male mice weight between 20 to 25 g were selected. After anesthesia, mice were fixed, and then the neck hair was shaved and sterilized. Then, the neck skin and muscle were split to expose Y‐shaped carotid artery. A slipknot was tied at the distal external carotid artery and a dead knot was tied at the near external carotid artery. Meanwhile, a slipknot was tied at the internal carotid artery and a slipknot was tied at the common carotid artery. After that, a cut was made between two knots of the external carotid artery longitudinally and a nylon monofilament suture (wire length 3 cm, diameter 0.102 mm, silicon diameter 0.22 ± 0.01 mm, CHASE RAY 0622) was inserted into the artery at the orientation of common carotid artery. Next, the external carotid artery between its two knots was cut off and the nylon monofilament suture was inserted into internal carotid artery. Then, the knot of external carotid artery was fastened. After above surgery procedure, the mice were set at a 37 °C incubator for 2 h. The nylon monofilament suture was pulled out and the knot at external carotid artery was fastened immediately. At the end, the knot at the common carotid artery was loosened. After sutured the wound, the mice were put at a 37 °C incubator until they wake up then a single antagonist (5 mg kg^−1^) or control was administered by gavage.

### Antibodies

The following primary antibodies and dilutions were used for immunostaining and western blotting: Brd4 (Abcam ab128874, Rabbit, 1:200); Brd4 (Invitrogen A301‐985A‐T, Rabbit, 1:500); Zo‐1 (Invitrogen 40‐2200, Rabbit, 1:500); Zo‐1 (Proteintech 21773‐1‐AP, Rabbit, 1:500); Claudin 5 (Invitrogen 352 500, Mouse, 1:500); Claudin 5 (Abclonal A10207, Rabbit, 1:500); GFAP (Sigma, G6171, Mouse, 1:1000); β‐catenin (Cell Signaling Technology, 8480s, Rabbit, 1:500); Fzd4 (Saribio, K109620P, Rabbit, 1:1 k); biotinylated IsolectinB4 (Vector Laboratories, B‐1205, 1:600); HA (Cell Signaling Technology, 3724s, Rabbit, 1:1 k); Flag (Sigma, F7425, Mouse, 1:2 k); 𝛽‐actin (Proteintech, 20536‐1‐AP, Rabbit, 1:10 000); 𝛽‐actin (Proteintech; 60008‐1‐Ig Mouse,1:2000); IgG (Bioss, bs‐0295p; Rabbit, 1:1 k). Flt4 (Abclonal A5605, Rabbit, 1:500); Flt1 (Proteintech, 13687‐1‐AP, Rabbit, 1:1 k).

Secondary antibodies: for immunostaining: DAPI (2 mg mL^−1^; Sigma; D9542); Alexa Fluor 488 Donkey Anti‐ Mouse IgG (Jackson 715‐547‐003, 1: 1 k), Cy3 Streptavidin (Jackson 016‐160‐084, 1: 1 k), Alexa Fluor cy5 Donkey Anti‐Rabbit IgG(Jackson 711‐175‐152, 1: 1 k); for Western blotting: Donkey anti‐Mouse 800 (LICOR 926‐32212, 1: 1 k); Donkey anti‐rabbit 680 (LICOR 926‐68023, 1: 1 k); and Donkey anti‐ mouse 680 (LICOR 926‐68072, 1: 1 k).

### Statistical Analysis

Data were present as mean ± SEM. Data normality of distribution were assessed before statistical analysis. The comparisons between two groups were analyzed by two‐tailed Student's *t*‐test. The comparisons among more than two groups were analyzed by ANOVA. All data were analyzed by GraphPad Prism 6 software. ns, not significant, ^*^ *p* < 0.05, ^**^ *p* < 0.01, ^***^ *p* < 0.001.

## Conflict of Interest

The authors declare no competing interests.

## Author Contributions

C.L., X.Z., J.X., and S.L. contributed equally to this work. C.L., J.J., and J.Z. conceived the experiments; X.Z. and Y.Z. helped with the construction of MCAO models; J.X. provided helps in IF staining; S.L. provided advices linking MS436 and stroke; Y.D. and F.J. helped in in vitro studies; S.L. and S.Z. provided helps in bioinformatics analysis; J.Z. and J.J. supervised the project and obtained funding support.

## Supporting information



Supporting Information

## Data Availability

The data that support the findings of this study are available from the corresponding author upon reasonable request.
