# Supplementary material for: Brd4 BD1 Domain Antagonism of MS436 Preserves Blood‐Brain Barrier Integrity via Rnf43/β‐Catenin Signaling Pathway
Source: Adv Sci (Weinh). 2025 Nov 20;13(7):e15584. doi: 10.1002/advs.202515584 (PMC12866839; doi:10.1002/advs.202515584)
Supplement: Supplementary file 1 — Supporting Information [file ADVS-13-e15584-s001.docx]

**Supplementary Information**

**Supplementary Figures**


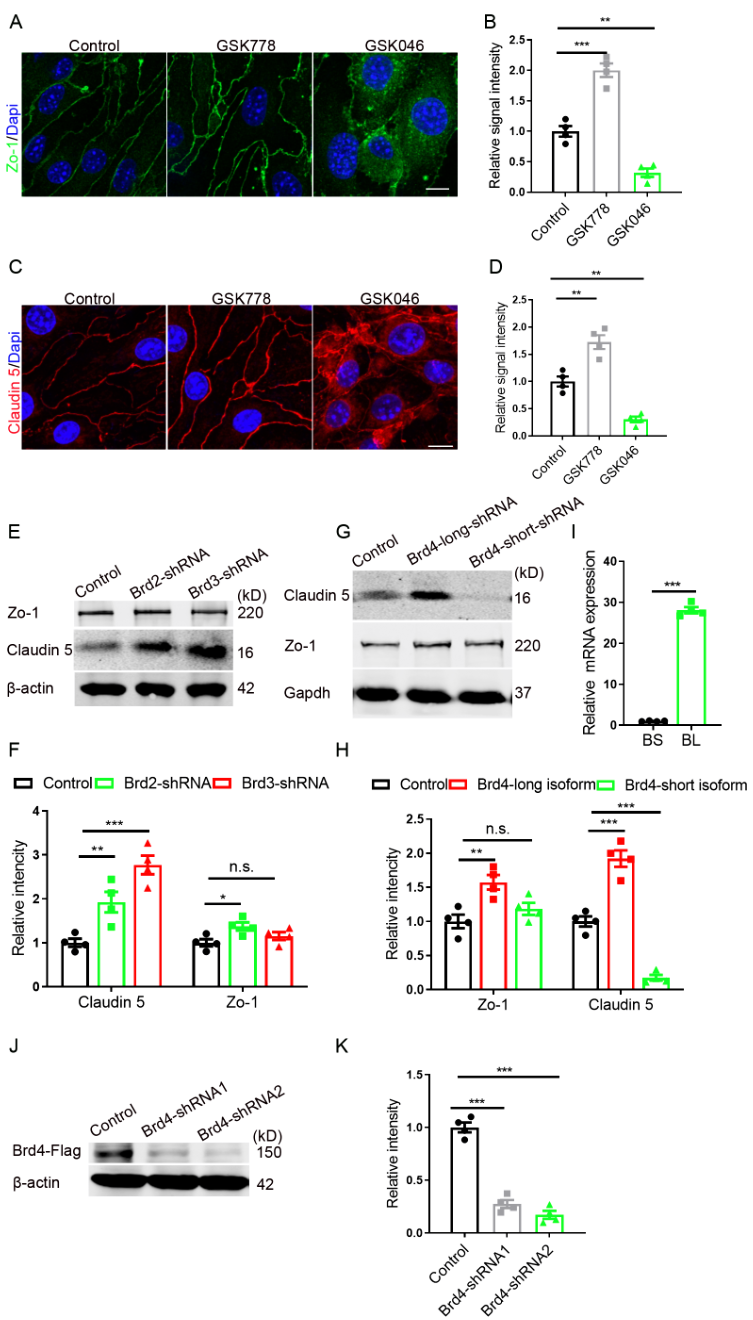


**Supplementary Figure 1. Brd4 is the main downstream effector of MS436**

(A) Regulation of Zo-1 by antagonists targeting BD domains (20 nM). Scale bar, 10 µm. (B) Statistic analysis of relative signal intensity, n=4. (C) Regulation of Claudin 5 by antagonists targeting BD domains (20 nM). Scale bar, 10 µm. (D) Statistic analysis of relative signal intensity, n=4. (E) Effects of the knockdown of BET family members on the level of TJ. (F) Statistic analysis of relative intensity of TJ in WB detection. n=4. (G) Effects of the knockdown of Brd4 isoforms on the level of TJ. (H) Statistic analysis of relative intensity of TJ in WB detection. n=4. (I) RT-PCR detection of Brd4 isoforms with pEC at E14.5. n=4. (J) Knockdown efficiencies detection for Brd4-shRNAs. (K) Statistic analysis of relative intensity of Brd4 in WB detection. n=4. Data were presented as mean±SEM, one-way ANOVA, Two-tailed Student’s t-test, n.s., no significant difference, *P < 0.05, **P < 0.01, ***P < 0.001.

**
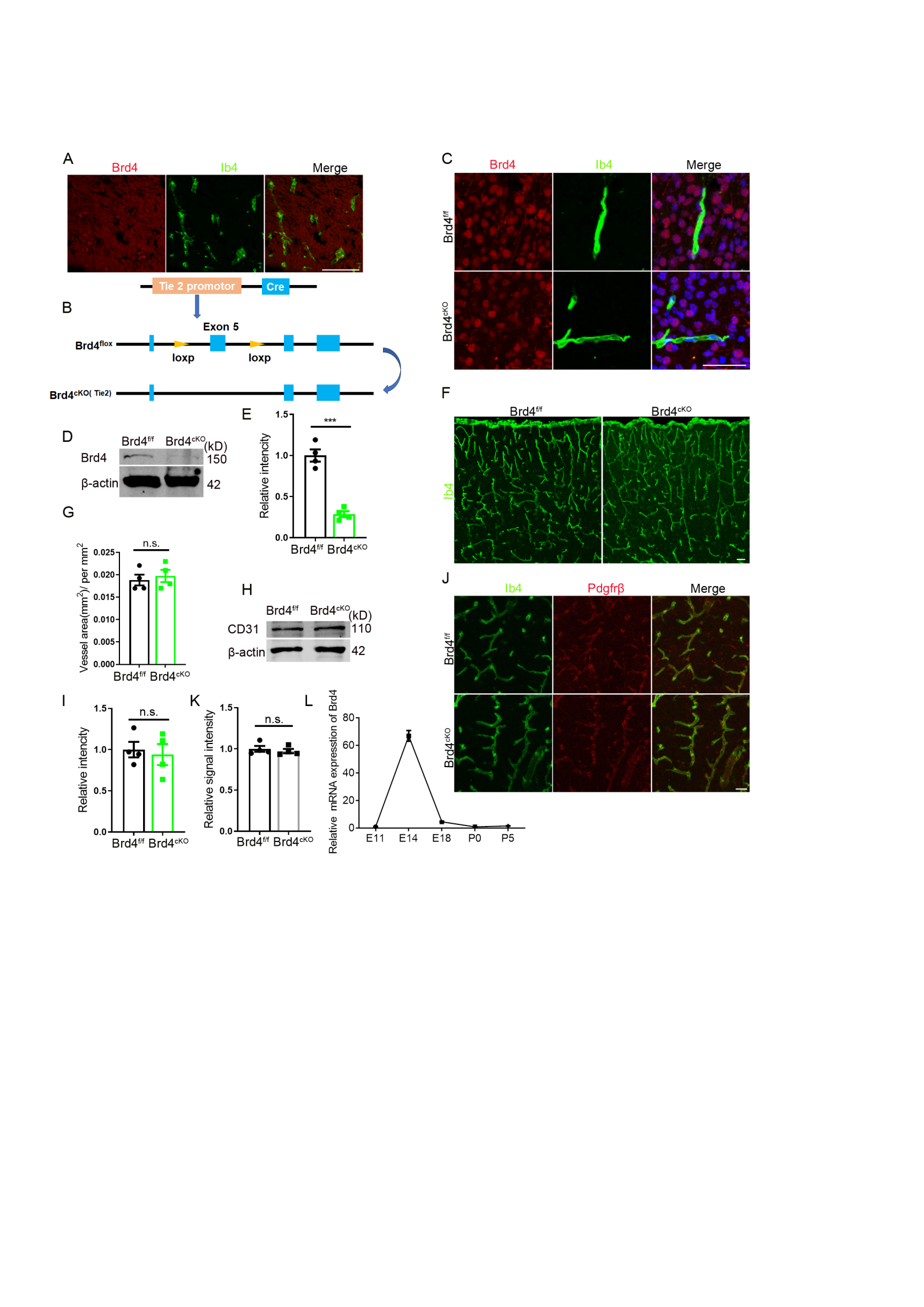
**

**Supplementary Figure 2. Brd4 is a negative regulator of the tight junction protein expression**

(A) Immunofluorescent staining for Brd4 with mice brain at E14. Scale bar, 50 µm. (B) Carton model of the construction strategy of Brd4 flox mice. (C) Immunofluorescent staining for Brd4 with mice brains at P0. Scale bar, 50 µm. (D) KO efficiency detection with pEC. (E) Statistic analysis of relative intensity of Brd4 in WB detection. n=4. (F) Immunofluorescent staining for Ib4 with mice brains at P5. Scale bar, 50 µm. (G) Statistic analysis of area of blood vessel, n=4. (H) WB detection for CD31 with mice cerebral cortex. (I) Statistic analysis of relative bond intensity of CD31 in WB detection. (J) Detection of pericytes recruitment. (K) Statistic analysis of relative signal intensity of Pdgfrβ. n=4. (L) Brd4 expression detection with endothelial cells taken from mice cerebral cortex. n=4. Data were presented as mean±SEM, one-way ANOVA, Two-tailed Student’s t-test, n.s., no significant difference, *P < 0.05, **P < 0.01, ***P < 0.001.

**
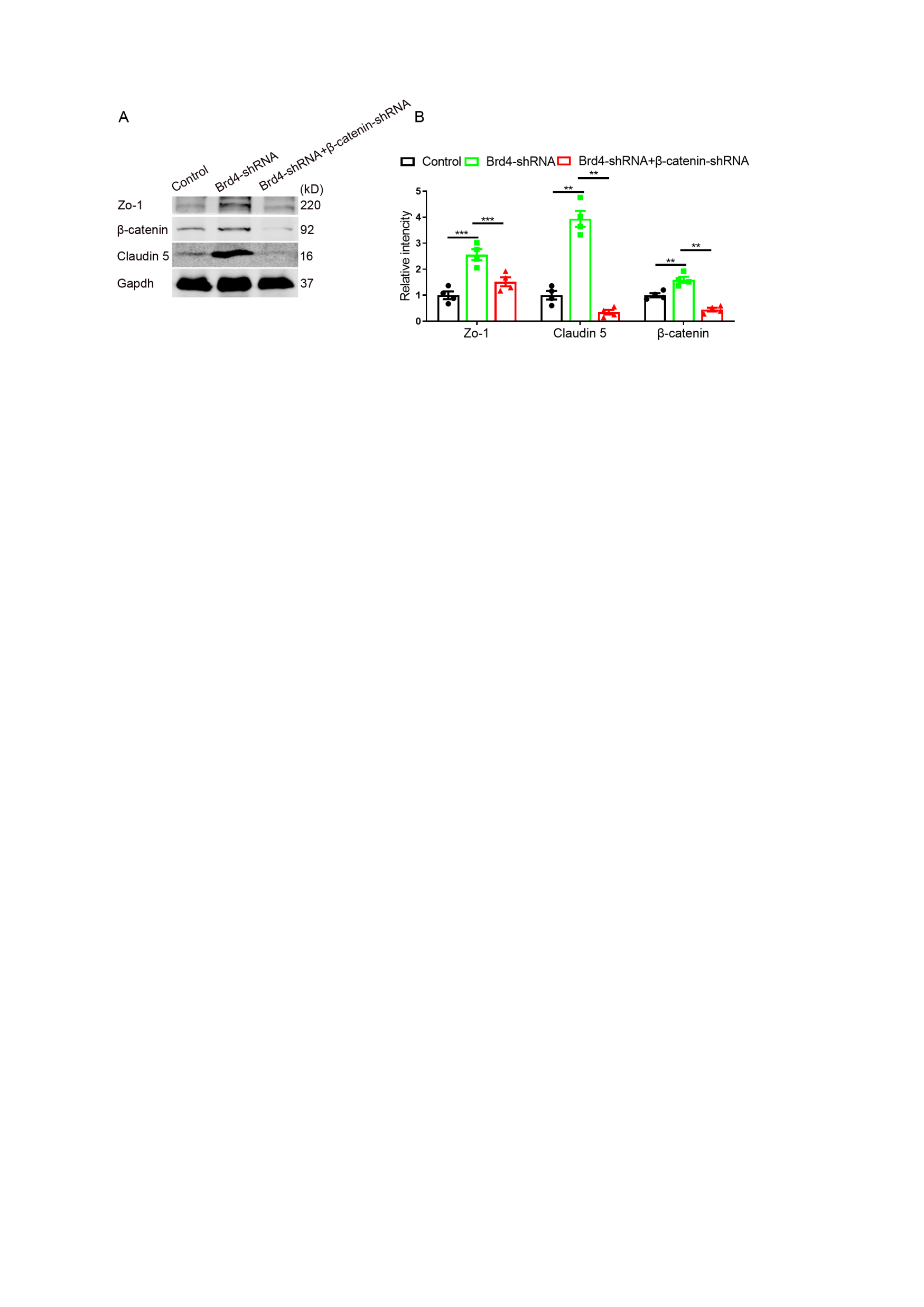
**

**Supplementary Figure 3. Knockdown of *β-catenin* could partially rescue the effects caused by loss of Brd4**

(A) Rescue assay by β-catenin knockdown with pEC. (B) Statistic analysis of relative intensity of TJ and β-catenin in WB detection. n=4. Data were presented as mean±SEM, one-way ANOVA, Two-tailed Student’s t-test, n.s., no significant difference, *P < 0.05, **P < 0.01, ***P < 0.001.


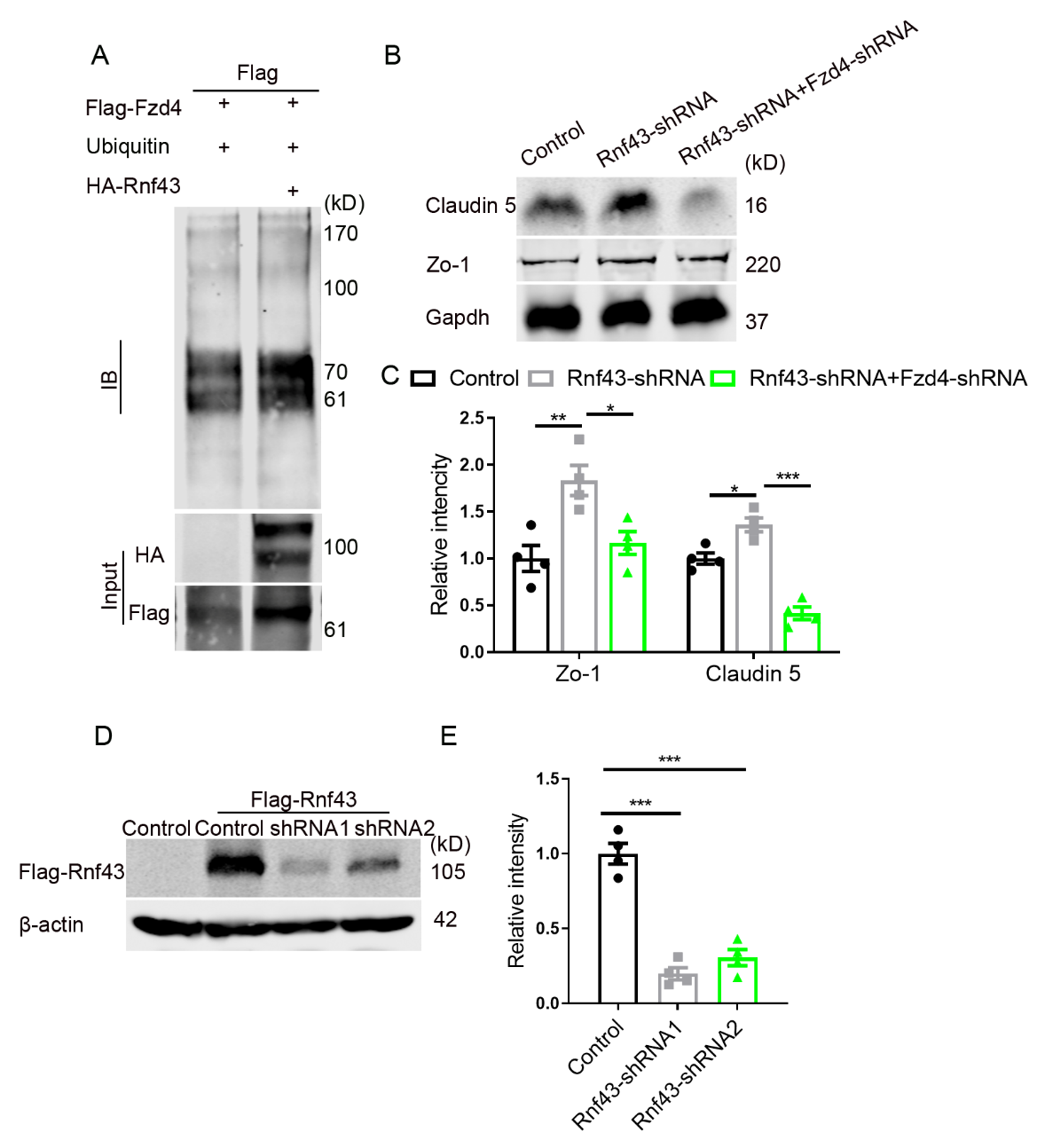


**Supplementary Figure 4. Rnf43 negatively regulates the expression of Fzd4**

(A) Rnf43 elevates the ubiquitination level of Fzd4. (B) Rescue assay by Fzd4 knockdown with pEC. (C) Statistic analysis of relative intensity of TJ in WB detection. n=4. (D) Rnf43 knockdown efficiencies detection with bEnd.3. (E) Statistic analysis of relative intensity of Rnf43 in WB detection. n=4. Data were presented as mean±SEM, one-way ANOVA, Two-tailed Student’s t-test, n.s., no significant difference, *P < 0.05, **P < 0.01, ***P < 0.001.


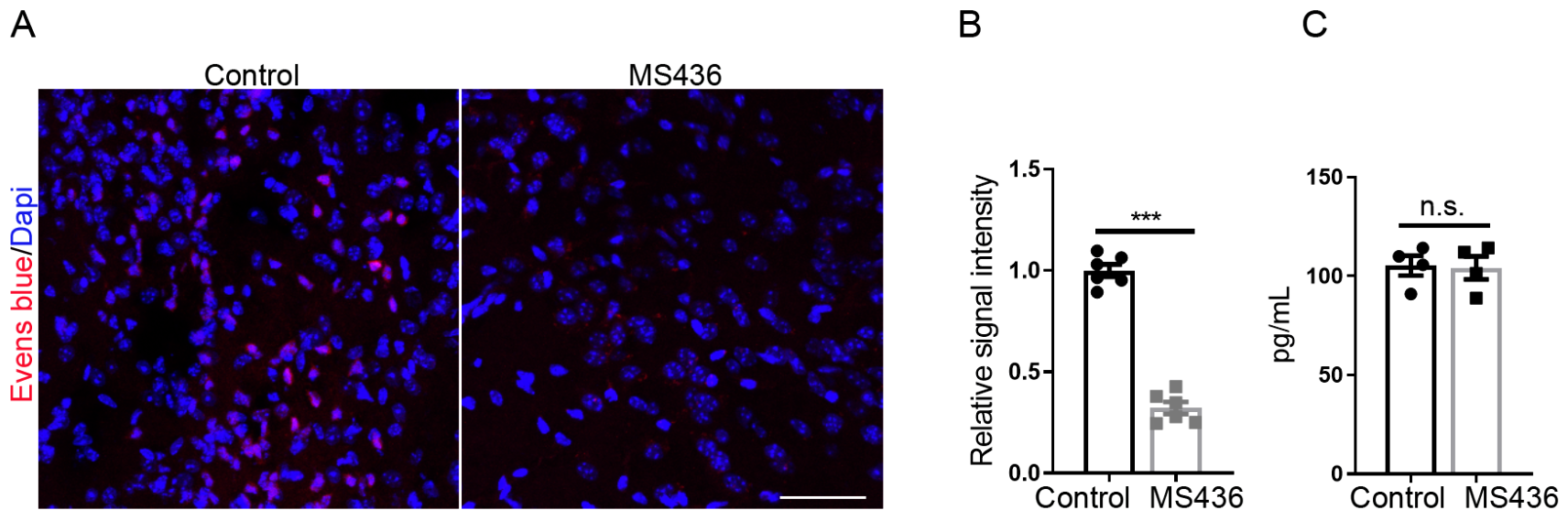


**Supplementary Figure 5. MS436 decreased the BBB leaky in stroke models**

(A) Evens blue staining with mice brain after stroke. (B) Statistic analysis of signal intensity of Evens blue. n=6. (C) Detection of serum Tnfα in stroke models. n=4. Data were presented as mean±SEM, one-way ANOVA, Two-tailed Student’s t-test, n.s., no significant difference, *P < 0.05, **P < 0.01, ***P < 0.001.

**Supplementary Table**

**Supplementary Table 1. Primers used in this study**

| *Brd4*- shRNA-1F | *TGCCATCTACACTACGAGAGTTCTCGAGAACTCTCGTAGTGTAGATGGCTTTTTC* |
| --- | --- |
| *Brd4*- shRNA-1R | *TCGAGAAAAAGCCATCTACACTACGAGAGTTCTCGAGAACTCTCGTAGTGTAGATGGCA* |
| *Brd4*- shRNA-2F | *TCCTCCCTGATTACTATAAGATCTCGAGATCTTATAGTAATCAGGGAGGTTTTTC* |
| *Brd4*- shRNA-2R | *TCGAGAAAAACCTCCCTGATTACTATAAGATCTCGAGATCTTATAGTAATCAGGGAGGA* |
| *Rnf43*- shRNA-1F | *TCGCAGCGGTTACTTGGCAGATCTCGAGATCTGCCAAGTAACCGCTGCGTTTTTC* |
| *Rnf43*- shRNA-1R | *TCGAGAAAAACGCAGCGGTTACTTGGCAGATCTCGAGATCTGCCAAGTAACCGCTGCGA* |
| *Rnf43*- shRNA-2F | *TACGCCCACTATCATCTTCCTTCTCGAGAAGGAAGATGATAGTGGGCGTTTTTTC* |
| *Rnf43*- shRNA-2R | *TCGAGAAAAAACGCCCACTATCATCTTCCTTCTCGAGAAGGAAGATGATAGTGGGCGTA* |
| *Fzd4*- shRNA-F | *TGGCTCCTCTCTTTACGTATTTCTCGAGGGCTCCTCTCTTTACGTATTTTTTTTC* |
| *Fzd4*- shRNA-R | *TCGAGAAAAAGGCTCCTCTCTTTACGTATTTCTCGAGGGCTCCTCTCTTTACGTATTTA* |
| *Fzd6*- shRNA-F | *TCCTAACCTGATGGGTCATTATCTCGAGATAATGACCCATCAGGTTAGGTTTTTC* |
| *Fzd6*- shRNA-R | *TCGAGAAAAACCTAACCTGATGGGTCATTATCTCGAGATAATGACCCATCAGGTTAGGA* |
| *Fzd10*- shRNA-F | *TTCGGCATGGCCAGCTCTTTATCTCGAGATAAAGAGCTGGCCATGCCGATTTTTC* |
| *Fzd10*- shRNA-R | *TCGAGAAAAATCGGCATGGCCAGCTCTTTATCTCGAGATAAAGAGCTGGCCATGCCGAA* |
| *β-catenin*-shRNA-F | *TCTGATATTGACGGGCAGTATCTCGAGATACTGCCCGTCAATATCAGTTTTTC* |
| *β-catenin*-shRNA-R | *TCGAGAAAAACTGATATTGACGGGCAGTATCTCGAGATACTGCCCGTCAATATCAGA* |
| *β-catenin*-F | *ATGGCTACTCAAGCTGACCTG* |
| *β-catenin*-R | *TTACAGGTCAGTATCAAACC* |
| *Fzd4*-F | *ATGGCCTGGCCGGGCACAGG* |
| *Fzd4*-R | *TTATACCACAGTCTCGTTGC* |
| *Rnf43*-F | *ATGAGTGGTGGCCACCAGCTG* |
| *Rnf43*-R | *TCACACAGCCTGCTCGCAC* |
| *pSicoR*-F | *TGTCAAAAAGGAAACTCACCC* |
| *pSicoR*-R | *GGCTATGAACTAATGACCCCGT* |
| *Brd4*-aa140-pt-F | *CAAATTGTTACATCTATttCAAGCCTGGAGATGACA* |
| *Brd4*-aa140-pt-R | *TGTCATCTCCAGGCTTGaaATAGATGTAACAATTTC* |
| *Brd4*-aa434-pt-F | *CCAACTGCTACAAGTACttCCCCCCTGACCATGAAG* |
| *Brd4*-aa434-pt-R | *CTTCATGGTCAGGGGGGaaGTACTTGTAGCAGTTGG* |
| *Rnf43*-12k-F | *AGGGGGCTGGAGTAGTTTCA* |
| *Rnf43*-12k-R | *GGACAGATGCAGAGGTCAGG* |
| *Rnf43*-11k-F | *TGCACAGCCCTAGCTACTTAT* |
| *Rnf43*-11k-R | *TAAACTGAGTCTTACTGACCCCAC* |
| *Rnf43*-10k-F | *TCAGTTCATTCCTAAAGACTTCCCA* |
| *Rnf43*-10k-R | *TCTGAAACAGGGTCTTACACAA* |
| *Rnf43*-5k-F | *CATTCGCGTTTCAGCACCTT* |
| *Rnf43*-5k-R | *ACCAAGACAAGAGTCTAGTTGGA* |
| *Rnf43*-2k-F | *GTCGCTCACTAGGGGGAGTA* |
| *Rnf43*-2k-R | *GGACTGCAATCACGCTCTCT* |
| *Rnf43*-1k-F | *GGCAGCATACTTTGTGGGTG* |
| *Rnf43*-1k-R | *CTTGAGGGGGCGGAGAATAA* |
